# Progression of Chronic Kidney Disease in Cats After Subcutaneous Ureteral Bypass Placement Compared to Cats With Idiopathic Chronic Kidney Disease

**DOI:** 10.1111/jvim.70242

**Published:** 2025-09-14

**Authors:** Zoe Bennett, Jack S. Lawson, Yu‐Mei Chang, Edward Shelton, Jonathan Elliott, Harriet M. Syme, Rebecca F. Geddes

**Affiliations:** ^1^ Clinical Science and Services Royal Veterinary College Hatfield UK; ^2^ Comparative Biomedical Sciences Royal Veterinary College London UK

**Keywords:** calcium oxalate, feline, renal recovery, ureterolithiasis

## Abstract

**Background:**

Chronic kidney disease (CKD) is a common sequela of ureteral obstruction, but many cats are non‐azotemic after subcutaneous ureteral bypass (SUB) placement.

**Objectives:**

Compare CKD progression rates after SUB placement with idiopathic CKD (iCKD), and explore variables associated with progression.

**Animals:**

Seventy‐one referred cats after SUB placement for ureteral obstruction and 89 primary care cats with iCKD.

**Methods:**

Retrospective observational longitudinal study. Baseline (3–6 months after SUB or at CKD diagnosis for iCKD cases) clinicopathological data and CKD progression (≥ 25% increase in creatinine concentration [Cr]) rates were compared between iCKD and SUB cats. Univariable logistic regression identified variables associated with SUB cat CKD progression.

**Results:**

Baseline Cr was lower in the SUB group (SUB, 2.0 mg/dL; iCKD, 2.3 mg/dL; *p* = 0.01). For SUB cats with a ≥ 25% increase in Cr within 1 year, 45% (9/20) had SUB obstruction. Of the remaining 11 cats, 35% had a positive urine culture. Only SUB blockage was associated with CKD progression in SUB cats (odds ratio, 33.33; confidence interval [CI], 3.80–292.60; *p* = 0.002). Progression of CKD within 1 year did not differ between groups (iCKD, 29.5%; SUB, 28.1%; *p* = 0.85), even after exclusion of obstructed cases (iCKD, 29.5%; SUB, 17.7%; *p* = 0.12). Median time to CKD progression was not different between groups (iCKD, 833 days; range, 21–2141; SUB, 653 days; range, 43–1662; *p* = 0.80).

**Conclusions and Clinical Importance:**

Progression of CKD after SUB placement occurs with similar frequency and time frame as in cats with iCKD, but should prompt assessment for SUB blockage and pyelonephritis.

**Abbreviations**
AKI: acute kidney injury
CI: confidence interval
Cr: whole blood, plasma or serum creatinine concentration
CKD: chronic kidney disease
FIV: feline immunodeficiency virus
HPF: high power field
iCKD: idiopathic chronic kidney disease
IRIS: International Renal Interest Society
NIBP: non‐invasive blood pressure
PCV: packed cell volume
RBCs: red blood cells
SDMA: symmetric dimethylarginine
SUB: subcutaneous ureteral bypass
USG: urine specific gravity
WBCs: white blood cells

## 1 Introduction

Ureteral obstruction, most commonly caused by ureterolithiasis [1, 2], is increasingly reported in cats. Obstruction of the ureter causes increased intrarenal pressure and subsequent decreased renal blood flow and glomerular filtration rate [3], consistent with an acute kidney injury (AKI). Recovery of kidney function after ureteral ligation in dogs is variable, with maximal return of function taking up to 4 months after 14 days of complete obstruction [4]. Glomerular filtration rate remained decreased compared with pre‐obstruction in these dogs, consistent with a diagnosis of chronic kidney disease (CKD). Partial ureteral obstruction appears to be associated with better recovery of kidney function in canine and murine surgical models [5, 6], and anecdotally a majority of cats with ureteral obstruction have a partial obstruction (Allyson Berent, personal communication). However, a substantial proportion of cats presenting with ureteral obstruction have pre‐existing CKD, and ureteral obstruction is the most common cause of acute‐on‐chronic kidney disease in cats [7].

In cats with ureteral obstruction, surgical management is recommended with a subcutaneous ureteral bypass (SUB) device or stent [8]. After SUB or ureteral stent placement, International Renal Interest Society (IRIS) stage at 3 months [9, 10, 11], and creatinine concentration (Cr) at one and 3 months [10], are significantly associated with survival time in cats. Additionally, kidney disease is the leading cause of death or euthanasia in cats after SUB placement [9, 10]. Therefore, CKD severity after AKI recovery appears to influence subsequent mortality in cats after ureteral obstruction.

Predictive factors of CKD progression and survival time in cats include age, proteinuria, anemia, serum phosphorus concentration, serum FGF23 concentration, Cr, and IRIS stage [12, 13, 14, 15]. However, the cats included in these studies generally were assumed to have idiopathic CKD (iCKD), with no identifiable inciting cause. Factors associated with CKD progression in cats after ureteral obstruction have not been explored.

Cats diagnosed with CKD are typically older, with a median age of 12.8–14.8 years [12, 14, 16], compared with a median age of 7–9 years for cats at the time of SUB device placement [1, 10, 17, 18]. Additionally, a large proportion of cats are non‐azotemic after SUB placement [19, 20]. Aging of the kidney in cats has been associated with progressive tubulointerstitial fibrosis [21] and in human medicine, aging is associated with decreasing glomerular filtration rate [22]. Maladaptive processes in the kidneys during aging are thought to impact the capacity for renal repair [23]. We therefore hypothesized that cats after SUB device placement might be less likely to have progressive CKD than cats with iCKD because of their younger age and the transient nature of their kidney insult.

Our aims were, firstly, to compare baseline clinicopathological variables associated with kidney disease in cats after placement of unilateral or bilateral SUB devices to cats with iCKD. Secondly, we sought to assess progressive CKD in cats after SUB placement and compare this progression to iCKD; and lastly, to identify factors that could predict CKD progression after SUB device placement.

## 2 Materials and Methods

Cats with a history of ureteral obstruction and SUB device placement were identified by retrospective review of clinical records at the Queen Mother Hospital for Animals, Royal Veterinary College (London, UK), between 1st January 2014 and 31st December 2019 and subsequently extended until 31st December 2022. Ethical approval for retrospective analysis of this anonymized data were not required by the Royal Veterinary College Ethics Review Committee. Owner consent for use of anonymized clinical data were obtained as part of the standard consent form for each visit to the hospital. The baseline visit was defined as the first re‐examination visit with a whole blood, plasma, or serum creatinine concentration (Cr) recorded between 3 and 6 months after SUB placement. Cases were excluded if they were on treatment for or developed hyperthyroidism, or did not have an appropriate visit within the baseline visit time limits. If a SUB blockage was identified on ultrasound‐guided SUB irrigation or fluoroscopic inspection, with a concurrent increase in Cr of ≥ 25% between discharge from the hospital after SUB placement and the baseline visit, the case was excluded. Juvenile cats with iatrogenic ureteral obstruction after ligation during ovariohysterectomy also were excluded.

Cats with a diagnosis of iCKD ≥ 9 years of age were identified by retrospective review of clinical records from two London‐based first opinion practices between 1 January 2014 and 31 December 2019. Ethical approval had been granted by the Royal Veterinary College Ethics Review Committee (URN 2013 1258E). Azotemic CKD was defined as Cr > 2 mg/dL (177 μmol/L) and concurrent urine specific gravity (USG) < 1.035 or Cr > 2 mg/dL (177 μmol/L) on two consecutive visits. A diagnosis of non‐azotemic CKD was defined as serum symmetric dimethyl arginine concentration (SDMA) > 14 μg/dL and Cr < 2 mg/dL. Cats were excluded if they were on treatment for or developed hyperthyroidism during follow‐up, were feline immunodeficiency virus (FIV) or feline leukemia virus positive, or had other clinically relevant concurrent disease. The baseline visit was defined as the date of diagnosis of CKD.

Data recorded for both groups included signalment and clinicopathological variables at baseline where available (Cr, IRIS stage, SDMA, serum phosphorus, total calcium and ionized calcium concentrations, packed cell volume [PCV], USG, urine pH, urine protein concentration [semi‐quantitative], urine protein: creatinine ratio, white blood cell [WBC] and red blood cell [RBC] numbers per high power field [hpf] on urine sediment examination, urine culture results, and systolic blood pressure). Creatinine concentration measurements from follow‐up visits were recorded. Where urine sediment examination reported a range for WBC or RBC per hpf, the midpoint of the range was used for analysis. If the cells were reported to be too numerous to count, they were recorded as 200/hpf. Positive urine cultures were recorded, but iCKD cats were not always cultured if urine sediment examination was inactive and therefore were assumed to have a negative urine culture. Serum or plasma biochemistry results were obtained from IDEXX reference laboratories for the iCKD cats and Royal Veterinary College reference laboratory or point‐of‐care analyzers (NOVA biomedical, CCX Blood Gas s/n Y0240301Z January 2014–September 2016; Radiometer ABL800 Flex Analyzer, October 2016‐study end and iSTAT 1, Abaxis VetScan 300 V, s/n 705971 May 2017‐study end) for the SUB cats, with the Royal Veterinary College laboratory reference laboratory used in preference where multiple measurements were available. Cases were assigned a diagnosis of hypertension if they were receiving or were prescribed anti‐hypertensive medication, had blood pressure ≥ 160 mmHg with retinal lesions, or had two systolic readings on concurrent visits of at least 7 days apart of ≥ 160 mmHg. Date of death was recorded; if the exact date was not known, the date of death was assigned as the 15th of that month.

Additional data recorded for the SUB cats included cause of the ureteral obstruction and presence of SUB blockage at any time during available follow‐up. Subcutaneous uretral bypass blockage was defined as a partial or complete SUB obstruction noted on the clinical record from imaging studies, with partial and complete considered equivalent. Ultrasound‐guided SUB irrigation was performed by a board‐certified specialist in diagnostic imaging, or residents in diagnostic imaging, surgery, or internal medicine, with suspect cases undergoing fluoroscopic inspection.

Progression of CKD was defined as an increase of Cr of ≥ 25% from the baseline Cr at any subsequent visit. The ≥ 25% cut‐off was considered a clinically relevant increase in Cr based on previous studies [12, 24]. Cats were followed until the last Cr measurement or progression. Cats were defined as stable if Cr remained within 25% of the baseline measurement. Cats were censored from the study if they were followed for < 12 months without documentation of a ≥ 25% increase in Cr. Cases that progressed after the 12‐month time point were defined as stable for the analysis of progression within 1 year. Blood and urine samples were obtained approximately every 3–4 months for both groups. The visits at which SUB cases demonstrated CKD progression were assessed regarding whether SUB blockage was implicated. Urine culture results at progression also were recorded for both groups.

Management recommendations for both iCKD and SUB cats after the baseline visit followed standard protocols. It was recommended that both groups be fed a renal diet once a diagnosis of azotemic CKD was identified. In the non‐azotemic SUB cats, a urinary diet was advised. If plasma phosphorus concentration remained above the IRIS stage target, aluminum hydroxide was prescribed along with the renal diet. Amlodipine was prescribed if systemic hypertension was diagnosed, with dose adjustment to maintain Doppler blood pressure < 160 mmHg. If ionized hypercalcemia developed on a renal diet, cats were transitioned to an early renal diet with subsequent addition of chia seeds.

### 2.1 Statistical Analysis

Statistical analysis was performed using IBM SPSS version 28 with significance at *p* < 0.05. Normality was assessed using the Shapiro–Wilk test and assessment of histograms. Numerical measures are reported as median (25th quartile, 75th quartile) with range reported for follow‐up times. For comparisons between baseline variables of the SUB cats and iCKD cats, the Mann–Whitney *U*‐test was used to assess non‐normally distributed continuous data. Chi squared or Fisher's exact tests were used to compare categorical data, with Kendall's tau *C* applied when assessing ordinal values.

Univariable binary logistic regression was used to assess for variables associated with progression in SUB cats, with all cats included and then with cats with documented SUB obstruction censored. Variables with more than 50% of missing data were not included.

A Kaplan–Meier curve with log‐rank test was performed using GraphPad Prism version 10.0.01 to analyze time to progression for all SUB and iCKD cats over their available follow‐up. Cats that did not progress were censored at the documented last Cr measurement. The SUB cats with a SUB blockage reported during available follow‐up were censored from this analysis.

## 3 Results

### 3.1 SUB Cats

A total of 199 cats had at least one SUB device placed during the study time frame. One hundred and twenty‐eight cats were excluded for the following criteria: iatrogenic ureteral obstruction at the time of neutering (*n* = 6), hyperthyroidism (*n* = 3), no suitable visit within the baseline visit time limits (*n* = 44), death before discharge or within 1 year without documentation of CKD progression (*n* = 45), lack of progression and inadequate follow‐up to define as stable at 12 months (*n* = 29) and a single cat that did not have a ureter at necropsy examination. Seventy‐one cats with SUB devices therefore were included in analyses.

Of these 71 cats, 25 were neutered males and 44 were spayed females, and one male and one female were recorded as intact. The most common breed was Domestic Shorthair (*n* = 37) followed by Ragdoll (*n* = 6), Domestic Long Hair (*n* = 5), Crossbreed (*n* = 5), Burmese (*n* = 3), Birman (*n* = 2), Persian (*n* = 2), Siamese (*n* = 2), Siberian (*n* = 2), and one each of the following breeds: Australian Mist, Bengal, British Shorthair, Devon Rex, Don Sphynx, Maine Coon, and Scottish Fold.

Bilateral SUB devices were placed in 25 cats, and 46 cats had a unilateral SUB device. Of the cats with unilateral SUBs, 41 were azotemic before SUB placement and five cats were non‐azotemic. Uroliths were diagnosed as the cause of the ureteral obstruction in 66 cats. One cat was reported to have a ureter obstructed by debris and blood clots, and in four cats the cause of obstruction was not clear from the clinical record.

The baseline visit was performed at a median of 121 days (range, 80–174) after unilateral or bilateral SUB implantation. Diet information at the baseline visit was available for 65 cats; 26 were receiving an over‐the‐counter diet for adult or senior cats, 30 were receiving a prescription renal diet, seven a prescription urinary diet, and two cats were receiving a hypoallergenic diet.

### 3.2 iCKD Cats

A total of 309 cats met the inclusion criteria for azotemic or non‐azotemic CKD. Two hundred and twenty cats were excluded on manual data review because of renal tumor (*n* = 1), FIV (*n* = 1), hyperthyroidism (*n* = 4), and lack of progression and inadequate follow up to define as stable at the 12‐month time period (*n* = 215). Eighty‐eight cats were included in the final data analysis.

Of these 88 cats, 42 were neutered males, 45 were spayed females, and one was an intact female. The most common breed was Domestic Shorthair (*n* = 65) followed by Domestic Long Hair (*n* = 8), Siamese (*n* = 4), Burmese (*n* = 3), Birman (*n* = 2), British Shorthair (*n* = 2), and one each of the following breeds: Bengal cross, Birman cross, Devon Rex, and Norwegian Forest. Diet information at the baseline visit was available for 82 cats; of these, 71 were receiving an over‐the‐counter diet for adult or senior cats, and 10 cats were receiving a prescription renal or early renal diet.

### 3.3 Baseline Variables Comparison Between SUB and iCKD Cats

At baseline, SUB cats were significantly younger than iCKD cats (SUB cats, 7.3 years; iCKD, 15.1 years; *p* = < 0.001; Table 1). Although kidney function appeared better in the SUB cats, with lower Cr (SUB cats, 2.0 mg/dL; iCKD, 2.3 mg/dL; *p* = 0.01) and higher USG (SUB cats, 1.022; iCKD, 1.017; *p* = < 0.001), the SUB cats had higher serum phosphorus concentrations (SUB cats, 4.5 mg/dL; iCKD, 4 mg/dL; *p* = < 0.001) and lower PCV (SUB cats, 30%; iCKD, 34%; *p* = < 0.001). The SUB cats had more active sediments with significantly more RBC per hpf (SUB cats, 150 RBC per hpf; iCKD, 3 RBC per hpf; *p* = < 0.001) and WBC per hpf (SUB cats, 15 WBC per hpf; iCKD, 0 WBC per hpf; *p* = < 0.001) and higher protein concentrations on the urine dipstick (SUB cats, 1+; iCKD, trace; *p* = < 0.001). Proportions of cats within each IRIS stage for the SUB and iCKD groups were significantly different (*p* = 0.02; Table 2).

**TABLE 1:** Comparison of baseline clinicopathological data for SUB and iCKD cats.

Variable	SUB median [25th, 75th percentiles]		iCKD median [25th, 75th percentiles]		*p*
	*n*		*n*		
Age (years)	71	7.3 [5.3, 10]	88	15.1 [12.5, 16.9]	**< 0.001**
Gender (% female)	71	37%	88	52%	0.20
Creatinine baseline (mg/dL)	71	2.0 [1.7, 2.5]	88	2.3 [2, 2.7]	**0.01**
SDMA baseline (μg/dL)	0^a^		59	17 [15, 20]	N/A
Phosphorus baseline (mg/dL)	58	4.5 [4.1, 5]	88	4 [3.5, 4.6]	**< 0.001**
Total calcium baseline (mg/dL)	35	9.9 [9.6, 10.5]	88	9.9 [9.5, 10.3]	0.54
Ionized calcium baseline (mmol/L)	38	1.39 [1.33, 1.42]	26	1.33 [1.27, 1.37]	**0.03**
PCV baseline (%)	57	30 [26, 34]	87	34 [32, 38]	**< 0.001**
Urine specific gravity baseline	66	1.022 [1.019, 1.029]	60	1.017 [1.015, 1.020]	**< 0.001**
Urine pH baseline	62	6 [5.5, 6]	60	5.5 [5, 6]	0.10
Urine protein baseline (semi‐quantative)	63	1+ [trace, 2+]	60	Trace [0, 1+]	**< 0.001**
WBC/hpf baseline	58	15 [4, 36]	25	0 [0, 3]	**< 0.001**
RBC/hpf baseline	64	150 [55, 200]	33	3 [0, 3]	**< 0.001**
Positive urine culture baseline (number positive/urine sediment performed)	69	13%	35	23%	0.26
Doppler NIBP baseline (mmHg)	16	143 [113, 154]	85	132 [120, 151]	0.47

*Note:* Bold values are statitistically significant (*P* < 0.05).

Abbreviation: NIBP, non‐invasive blood pressure.

^a^ SDMA had not been routinely measured in the SUB cats.

**TABLE 2:** Number and proportion of cats in each of the IRIS CKD stages for SUB and iCKD cats at baseline.

IRIS stage	SUB *n*	% of SUB cats	iCKD *n*	% of iCKD cats
1	13	18%	5	6%
2	50	71%	65	74%
3	8	11%	16	18%
4	0	—	2	2%

*Note:* Chi‐squared analysis found the proportions of cats within each IRIS stage for SUB and iCKD cats were significantly different (*p* = 0.02).

A higher proportion of SUB cats were non‐azotemic at baseline (34 SUB cats, 47.8%; 21 iCKD cats, 23.8%; *p* = 0.002). No significant difference was found between the presence of a positive urine culture within 1 year of baseline (14/71, 19.7% SUB; 14/65, 21.5% iCKD; *p* = 0.83) or of a diagnosis of hypertension within the same timeframe (5/35, 14.3% SUB; 18/88, 20.5% iCKD; *p* = 0.46). Baseline blood pressure was not significantly different between groups (143 mmHg SUB; 132 mmHg iCKD, *p* = 0.47); but at baseline, 3/71 (4.2%) SUB cats were receiving amlodipine compared with 9/88 (10.2%) iCKD cats. Additionally, no SUB cats were diagnosed with hypertension at baseline, but 4/88 (4.5%) iCKD cats were hypertensive. One SUB cat had multiple concurrent blood pressure readings > 160 mmHg and was included in the hypertensive group by this criterion, but was clinically suspected to have situational hypertension.

### 3.4 Progression of CKD Within 12 Months of Baseline

Of the 88 iCKD cats included in the study, 26 cats (29.5%) had progression of their CKD within 12 months of the baseline visit, with the remaining 62 cats having stable kidney function. Of the 26 cats with progressive CKD, four had positive urine cultures at the time of progression (15.3%), 14 cats had inactive urine sediment, and culture was not performed, and eight cats had no urinalysis obtained at the visit documenting progression. Of the non‐progressive CKD cats, eight of 62 (12.9%) had positive cultures at baseline or in the next 12 months.

Of the 71 SUB cats, 20 (28.1%) had progression of their CKD within 12 months of baseline. No significant difference was found in the proportion of cats with CKD progression between the iCKD and SUB groups (iCKD, 29.5%; SUB, 28.1%; *p* = 0.85). Of the 20 SUB cats that demonstrated CKD progression within 12 months, 11 had unilateral SUBs and nine had bilateral SUBs. Nine of 20 SUB cats had SUB blockage documented on imaging at the time of CKD progression; three of these had unilateral SUBs and six had bilateral SUBs.

### 3.5 Factors Associated With CKD Progression in SUB Cats Within 12 Months of Baseline

In univariable analysis, SUB blockage was found to be associated with increased odds of CKD progression (odds ratio, 33.33; confidence interval [CI], 3.80–292.60; *p* = 0.002) in SUB cats (Table 3). When cats with SUB obstruction were excluded, no variables were associated with CKD progression (see Table S1).

**TABLE 3:** Clinicopathological variables assessed for association with CKD progression in all SUB cats.

Variable	Category	Number of cats in analysis		OR	95% CI	*p*
		Stable	Progressive			
Sex (female)		51	20	1.10	0.37–3.25	0.86
Age at baseline (years)		51	20	1.01	0.86–1.18	0.94
Bilateral, unilateral azotemic, or unilateral non‐azotemic ureteral obstruction	Bilateral	16	9			0.74
	Unilateral azotemic	30	11	0.65	0.22–1.90	
	Unilateral non‐azotemic	5	0	0	0	
SUB position (unilateral)		51	20	1.79	0.62–5.17	0.28
Creatinine baseline (mg/dL)		51	20	1.53	0.72–3.24	0.27
IRIS stage	1	10	3			0.78
	2	36	14	1.30	0.31–5.42	
	3	5	3	2	0.29–13.74	
Phosphorus baseline (mg/dL)		42	16	1.88	0.75–4.69	0.18
Total calcium baseline (mg/dL)		25	10	0.52	0.19–1.42	0.20
Ionized calcium baseline (mmol/L) × 10		26	12	1.15	0.54–2.47	0.72
PCV baseline (%)		40	13	0.89	0.80–1.00	0.05
Urine specific gravity baseline × 1000		47	19	1.00	0.93–1.07	0.96
Urine pH baseline	5, 5.5	16	10			0.57
	6, 6.5	21	7	0.53	0.17–1.71	
	7, 7.5, 8	8	0	0	0	
Urine dipstick protein baseline	0, trace	11	5			0.175
	1+	15	9	1.32	0.35–5.05	
	2+, 3+	20	3	0.33	0.07–1.65	
WBC/hpf baseline	< 10	20	5			0.29
	10–49	13	9	2.77	0.76–10.13	
	≥ 50	7	4	2.29	0.48–11.00	
RBC/hpf baseline	< 50	10	4			0.08
	50–199	10	9	2.25	0.52–9.77	
	≥ 200	25	5	0.5	0.11–2.25	
Positive urine culture at baseline		50	19	1.38	0.31–6.16	0.68
Positive urine culture within 1 year		51	20	1.56	0.45–5.39	0.49
SUB obstruction within 1 year		51	20	33.33	3.80–292.60	0.002

### 3.6 Progression of CKD Within 12 Months of Baseline for SUB Cats With Obstructed SUBs Excluded

Cats with SUB blockage at the time of progression were censored from subsequent analysis, leaving 11 of 62 (17.7%) cats with CKD progression. One SUB cat was documented to have a SUB blockage within 12 months of baseline, but this blockage was not associated with CKD progression (because the ureter was patent) and this case was included as a stable case in further analysis. The proportions of cats with progression according to IRIS stage for both groups are presented in Table 4. Of the 11 SUB cats with non‐obstructive CKD progression, four cats had positive urine cultures (36.4%), seven cats had negative urine cultures, including one that had a negative culture but was receiving antibiotics, and one cat was clinically suspected to have pyelonephritis but was culture negative. Of the non‐progressive SUB cats, nine of 51 (17.6%) had positive cultures at baseline or in the next 12 months. No difference was found in the proportion of cats demonstrating CKD progression between the iCKD and SUB groups after censoring of cats with SUB blockage (iCKD, 29.5%; SUB, 17.7%; *p* = 0.12).

**TABLE 4:** CKD progression within 1 year by IRIS stage for iCKD and SUB cats (excluding cats with SUB blockage).

IRIS stage	SUB		iCKD	
	Total *n*	Progressive *n* (%)	Total *n*	Progressive *n* (%)
1	13	3 (23)	5	2 (40)
2	43	7 (16)	65	18 (28)
3	6	1 (17)	16	5 (31)
4	—	—	2	1 (50)

Of the 11 SUB cats that progressed within 12 months of baseline without SUB blockage, the median (25th, 75th percentiles) Cr at the time progression was first documented was 3 mg/dL (2.6, 4.4), which was an increase of 50% (29, 100) from baseline Cr 1.8 mg/dL (1.6, 2.6). For reference, the baseline median Cr for the non‐progressive cats was 2.0 mg/dL (1.7, 2.5).

### 3.7 Long Term Follow‐Up Data

The iCKD cats were followed for a median of 623 days (range, 49–2465) to progression or last Cr measurement. Over the entire follow‐up period, 48/88 (55%) iCKD cats demonstrated CKD progression. By IRIS stage at baseline, progression occurred in 3/5 IRIS stage 1 cats (60%), 37/65 IRIS stage 2 cats (57%), 7/16 IRIS stage 3 cats (44%) and 1/2 of the IRIS stage 4 cats (50%).

Over the available follow‐up, 26/71 (37%) SUB cats had documented SUB blockage. When these cats were excluded, the remaining 45 SUB cats were followed for median of 575 days (range, 43–1692) to CKD progression or last Cr measurement. Of these 45 cats, 27 (60%) demonstrated CKD progression. By IRIS stage at baseline, progression occurred in 7/10 IRIS stage 1 cats (70%), 17/30 IRIS stage 2 cats (57%) and 3/5 IRIS stage 3 cats (60%).

**FIGURE 1:**
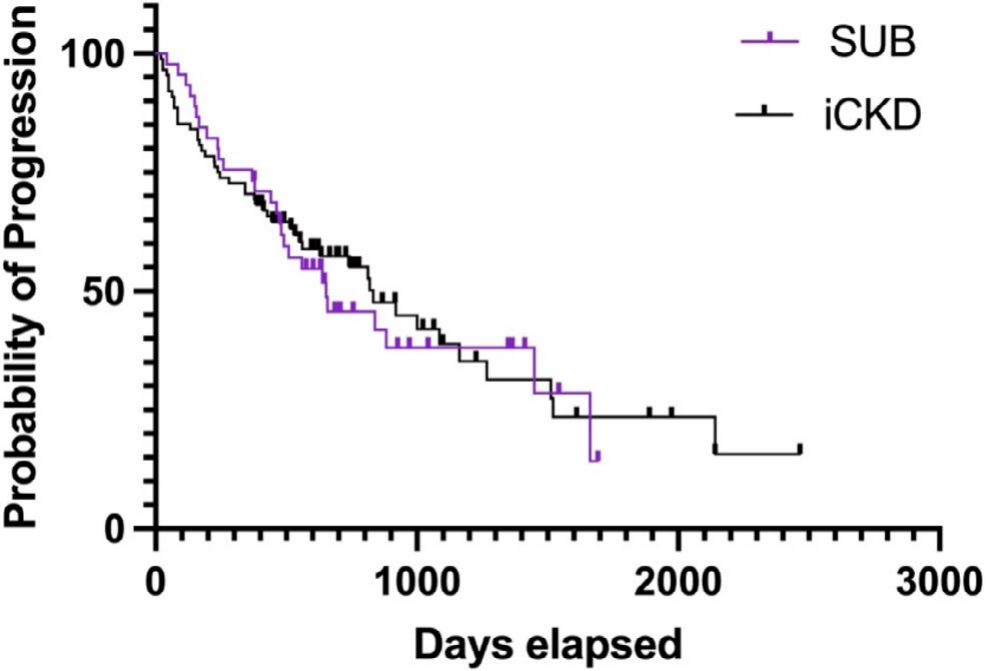
SUB (purple) compared to iCKD (black) time to progression with cases censored at the date of the last visit. When compared using the log rank test no significant difference in time to progression was observed between iCKD cats and SUB cats (iCKD median, 833 days; range, 21–2141; SUB cats, median, 653 days; range, 43–1662; *p* = 0.80).

No significant difference was found in the time to progression between the iCKD cats and SUB cats without SUB blockage (iCKD median, 833 days; range, 21–2141; SUB cats median, 653 days; range, 43–1662; *p* = 0.80; Figure 1).

### 3.8 Death

Twenty‐two of 71 (31%) SUB cats died or were euthanized; of those known to be dead, the median time from baseline to death was 516 days (range, 74–1468), with three deaths in the first 12 months. Sixty‐six of 88 (75%) iCKD cats were known to be dead a median of 741 days from baseline (range, 50–2470), with 10 deaths in the first 12 months.

## 4 Discussion

We found that CKD progression occurred in cats after SUB placement with similar frequency and time frame as cats with iCKD when compared both within 1 year and during all available follow‐up. However, in 45% of SUB cats documented to have a ≥ 25% increase in Cr, obstruction of the SUB device occurred. Obstruction of the SUB was the only variable significantly associated with CKD progression within 12 months based on univariable logistic regression analysis. Although it appears that SUB cats have CKD analogous to iCKD cats with regard to a propensity for progression, we found significant differences in several variables at baseline, including lower PCV, higher ionized calcium concentration, and more active urine sediment despite similar rates of positive urine culture. Of the SUB cats demonstrating CKD progression within 1 year without SUB obstruction, 36% had positive urine cultures.

Obstruction of the SUB device was significantly associated with a ≥ 25% increase in Cr, which was identified in 45% of the SUB cats with progression. Obstruction of the SUB device is a frequently reported occurrence that can occur secondary to blood clots, kinking, or calcification of the SUB, with calcification reported in 24% of SUB devices with long‐term follow‐up [10]. When obstruction occurs in association with increased Cr, and the obstruction cannot be relieved by irrigating the device, SUB replacement generally is recommended. Tetrasodium EDTA helps resolve SUB obstruction [25] and might prevent obstruction from developing [26]. Our findings suggest that evaluation of the SUB device for obstruction is crucial when a clinically relevant increase in Cr is documented, and emphasizes the ongoing need to pre‐emptively irrigate SUB devices to maintain patency.

Interestingly, even when cats with SUB obstruction were censored, no significant difference was identified in the proportion of cats demonstrating CKD progression during the first 12 months from baseline, or during the entire available follow‐up, between SUB cats and cats with iCKD (Figure 1). This finding did not support our hypothesis that the SUB group would be more likely to remain stable because they were younger, had undergone an acute post‐renal insult with subsequent renal pelvic decompression, and often have non‐azotemic CKD after SUB placement. Although magnitude of azotemia repeatedly has been associated with survival in cats with CKD [12, 13, 14, 15], the ability of Cr to predict CKD progression in cats has been inconsistent among studies [12, 15]. In our study, Cr was not associated with the risk of CKD progression in SUB cats. Furthermore, for both groups, the proportion of cats that demonstrated CKD progression was similar across IRIS stages. Idiopathic CKD in cats most commonly is characterized by tubulointerstitial fibrosis on histopathology, with more severe fibrosis associated with more advanced IRIS stage [27]. Tubulointerstitial fibrosis is the common outcome of most progressive kidney diseases in cats as well as in other species and has been documented in aging cats without kidney disease [28], experimentally‐induced ischemia [29] and urethral obstruction [30]. Although the histopathology of kidneys from cats after ureteral obstruction is not reported, tubulointerstitial fibrosis likely would be present. Although CKD progression is likely multifactorial, a common underlying pathology could explain the lack of difference in progression between iCKD and SUB cats, despite the difference in signalment.

Other than SUB obstruction, none of the included variables was found to be associated with CKD progression in the cohort of SUB cats in our study. This finding was surprising because proteinuria and Cr are associated with a subsequent diagnosis of azotemic CKD in older cats [31]. However, many biomarkers have limitations during the early stages of CKD in cats [32], and 48% of the SUB cats assessed for CKD progression in our study were non‐azotemic. Because of the low number of cats with progression, it is possible that our study was underpowered to identify additional variables associated with CKD progression. Additional larger studies are required to identify predictive variables for CKD progression in SUB cats that could be manipulated to improve prognosis.

In humans, the presence of an internal ureteral stent for > 3 months is associated with deterioration in kidney function in 50% of patients, with this decline being associated with positive urine culture, a baseline estimated glomerular filtration rate (eGFR) of < 60 mL/min, or extraluminal obstruction [33]. Additionally, in human patients in whom a stent remains in place longer than recommended, a significant proportion develop CKD [34]. In contrast, we did not find any evidence that the presence of a SUB device increased the risk of CKD progression because rates of progression did not differ from cats with iCKD. This finding was despite the fact that many cats with ureteral obstruction already have concurrent CKD based on the ultrasonographic appearance of their kidneys [1, 35, 36], or documentation of pre‐existing CKD in the history [9]. In our study, it was difficult to assess prior history of CKD, and imaging characteristics were not assessed for evidence of prior CKD. Nevertheless, the presence of pre‐operative azotemia in 89% of cats with unilateral ureteral obstruction in our study, indicating the presence of pre‐existing disease in the contralateral kidney, was not found to be associated with future CKD progression.

The SUB cats had significantly lower PCV compared with the iCKD cats, despite having less advanced CKD and being over 3 months beyond surgery. A lower PCV at discharge has negative associations with survival in cats after ureteral stent placement [11], but PCV was not found to be associated with CKD progression in our study. Most of the SUB cats were sedated for SUB irrigation at the time of the baseline visit, and because of the retrospective nature of our study, it was not possible to know if laboratory testing was performed during sedation. Sedation and anesthesia can cause a decreased PCV, although variably replicated in studies [37, 38, 39], and a sedation protocol using an opioid and alpha‐2 agonist similar to that used for SUB irrigation found no significant change in PCV in healthy cats [40]. Although anemia can be a consequence of CKD because of decreased erythropoietin production, this finding would be unexpected in IRIS stage 1–2 cats [41], which constitutes the majority of the SUB cats in our study. Therefore, if the lower PCV in the SUB cat group was unrelated to sedation, the anemia could reflect ongoing blood loss associated with the SUB device, systemic inflammation triggered by the presence of the implants, or a sequela of the recent hospitalization and surgery performed. Notably, the SUB cats had significantly more RBCs on urine sediments in our study, which might have contributed to a lower PCV in these cats.

A more active urinary sediment (RBCs, WBCs, and protein) was identified in the SUB cats compared with iCKD cats. This finding is considered consistent with the propensity for sterile cystitis in cats after SUB placement [18]. In humans, significant bladder wall inflammation has been identified in patients with indwelling ureteral stents [42], and it is widely accepted that stents are associated with sterile pyuria [43]. Equivalent histopathology from bladders of cats is lacking, but importantly, SUB and iCKD cats had equivalent rates of positive urine cultures at baseline and during the following 12 months, suggesting that the presence of a SUB device does not increase the risk of bacteriuria compared to cats with iCKD. Although rates of infection might be similar, urinary tract infections can be problematic in cats with SUB devices because of the difficulty of clearing infection when an implant is present as a nidus for biofilm formation, as reported in humans with ureteral stents [44]. A positive urine culture was documented at the time of CKD progression in 15% of iCKD cats and 36% of SUB cats in our study. These cats might have had pyelonephritis that contributed to their increased Cr, but the retrospective nature of our study makes it difficult to confirm in all cases. The possibility of bacterial infection in a SUB device should be investigated in a cat with progressive azotemia, and pyelonephritis should remain a carefully considered differential diagnosis for these cases.

Interestingly, despite less advanced CKD and a higher proportion of cats being fed a phosphate‐restricted renal diet, the SUB cats had significantly higher serum phosphorus concentrations at baseline compared with the iCKD cats. The clinical relevance of this finding is uncertain because serum phosphorus concentration was not associated with the risk of progression in our study. The total calcium concentration was similar between the SUB and iCKD cats at baseline, but the ionized calcium concentration was significantly higher in the SUB cats. This finding is consistent with the findings of a previous study in which cats with CKD and upper urinary tract uroliths had higher whole blood ionized calcium concentration than those without urolithiasis [45].

Surprisingly, a diagnosis of hypertension within 12 months was equally prevalent in the iCKD and SUB cats, despite the disparity in age between the groups, and given that the prevalence of hypertension increases over time in cats with CKD [46]. Hypertension is commonly detected in cats with iCKD, and treatment is recommended because of the risk of end organ damage [47, 48, 49]. Our results emphasize the importance of monitoring blood pressure as well as performing fundic examination to assess for hypertensive lesions in all cats with CKD, including cats after SUB placement. Proteinuria based on semiquantitative analysis was not identified as a predictor of CKD progression in SUB cats in our study. Use of dipstick protein measurement was a limitation of our study, and it is possible that an association might have been found if urine protein‐to‐creatinine ratio was used instead. However, this test is not performed routinely at our institution for SUB cats because of the difficulty in interpreting results in the presence of the implant, which might contribute to urinary tract inflammation, as discussed above.

Our study had several limitations based on its retrospective design, missing data, and number of cases lost to follow‐up. The SUB and iCKD cats had Cr measurements performed at different laboratories, and in‐house analyzer data were included when reference laboratory data were not available. Despite different analyzers being used, every effort was made to ensure that Cr measurements within groups were made using the same analyzer. Urine culture was only performed in iCKD cats if bacteriuria was documented on urine sediment examination, and therefore, underestimation might have occurred in this group. Because of limitations in the clinical records of referral patients, a prior diagnosis of CKD in SUB cats remains possible and could have affected their risk of future CKD progression. Cats with SUB obstruction documented at the time of progression based on Cr were excluded from some additional analyses, but it remains possible that a proportion of these cats had patent ureters and, therefore, true progression of their kidney disease.

The data from our study supports ongoing management of SUB cats in a manner analogous to cats with CKD, based on recommendations for their current IRIS stage. Should an increase in Cr be detected, further assessment should be performed to exclude SUB obstruction or obstruction of the contralateral ureter, and urine culture should be performed to assess for bacteriuria that could indicate pyelonephritis. Additional studies are required to explore predictive variables of CKD progression that are modifiable so as to improve survival of cats after SUB placement. However, our finding that CKD has similar progression rates in cats after SUB placement compared to cats with iCKD remains an important finding, considering that SUB placement is a salvage procedure and that cats with iCKD can have prolonged survival [14].

## Disclosure

Authors declare no off‐label use of antimicrobials.

## Ethics Statement

Approved by Royal Veterinary College (RVC) Ethics Review Committee (URN 2013 1258E). Authors declare human ethics approval was not needed.

## Conflicts of Interest

The authors declare no conflicts of interest.

## Supporting information


**Table S1:** Clinicopathological variables assessed for association with CKD progression in SUB cats with obstructed SUBs excluded.
