# Supplementary material for: Progression of Chronic Kidney Disease in Cats After Subcutaneous Ureteral Bypass Placement Compared to Cats With Idiopathic Chronic Kidney Disease
Source: J Vet Intern Med. 2025 Sep 14;39(5):e70242. doi: 10.1111/jvim.70242 (PMC12434176; doi:10.1111/jvim.70242)
Supplement: Supplementary file 1 — Table S1: Clinicopathological variables assessed for association with CKD progression in SUB cats with obstructed SUBs excluded. [file JVIM-39-e70242-s001.docx]

|  |  | **Number of cats in analysis** | |  |  |  |
| --- | --- | --- | --- | --- | --- | --- |
| **Variable** | **Category** | **Stable** | **Progressive** | **OR** | **95% CI** | **p value** |
| Sex (female) |  | 51 | 11 | 0.71 | 0.19-2.66 | .61 |
| Age at baseline (years) |  | 51 | 11 | 1.06 | 0.88-1.29 | .53 |
| Bilateral, unilateral azotemic or unilateral non-azotemic ureteral obstruction | Bilateral | 16 | 3 |  |  | .89 |
|  | Unilateral azotemic | 30 | 8 | 1.42 | 0.33-6.12 |  |
|  | Unilateral non-azotemic | 5 | 0 | 0 | 0 |  |
| Creatinine baseline (mg/dl) |  | 51 | 11 | 0.82 | 0.29-2.37 | .72 |
| IRIS stage | 1 | 10 | 3 |  |  | .85 |
|  | 2 | 36 | 7 | 0.65 | 0.14-2.97 |  |
|  | 3 | 5 | 1 | 0.67 | 0.05-8.16 |  |
| Phosphorus baseline (mg/dl) |  | 42 | 9 | 2.4 | 0.67-8.66 | .18 |
| Ionised calcium baseline (mmol/L) x 10 |  | 26 | 8 | 1.34 | 0.57-3.14 | .50 |
| PCV baseline (%) |  | 41 | 9 | 0.94 | 0.82-1.07 | .36 |
| Urine specific gravity baseline x 1000 |  | 47 | 11 | 1.01 | 0.92-1.11 | .80 |
| Urine pH baseline | 5, 5.5 | 16 | 5 |  |  | .98 |
|  | 6, 6.5 | 21 | 6 | 1.14 | 0.28-4.74 |  |
|  | 7, 7.5, 8 | 8 |  | 0 | 0 |  |
| Urine dipstick protein baseline | 0, trace | 11 | 3 |  |  | .19 |
|  | 1+ | 15 | 6 | 1.47 | 0.30-7.19 |  |
|  | 2+, 3+ | 20 | 1 | 0.18 | 0.02-1.98 |  |
| WBC/hpf baseline | <10 | 20 | 1 |  |  | .14 |
|  | 10-49 | 13 | 6 | 9.23 | 0.99-85.78 |  |
|  | ≥50 | 7 | 3 | 8.57 | 0.76-96.52 |  |
| RBC/hpf baseline | <50 | 10 | 3 |  |  | .34 |
|  | 50-199 | 10 | 4 | 1.33 | 0.24-7.56 |  |
|  | ≥200 | 25 | 3 | 0.4 | 0.07-2.33 |  |
| Positive urine culture at baseline |  | 50 | 10 | 1.83 | 0.31-10.75 | .50 |
| Positive urine culture within 1 year |  | 51 | 11 | 2.67 | 0.64-11.08 | .18 |

Table S1 – Clinicopathological variables assessed for association with CKD progression in SUB cats with cats with obstructed SUBs excluded.
